# Evaluating different methods of MR-based motion correction in simultaneous PET/MR using a head phantom moved by a robotic system

**DOI:** 10.1186/s40658-022-00442-6

**Published:** 2022-03-03

**Authors:** Eric Einspänner, Thies H. Jochimsen, Johanna Harries, Andreas Melzer, Michael Unger, Richard Brown, Kris Thielemans, Osama Sabri, Bernhard Sattler

**Affiliations:** 1 Clinic of Radiology and Nuclear Medicine, Magdeburg, Germany; 2 Department of Nuclear Medicine, Leipzig University Hospital, Leipzig, Germany; 3 Innovation Center Computer Assisted Surgery (ICCAS), Faculty of Medicine, University Leipzig, Leipzig, Germany; 4 Institute of Nuclear Medicine, University College London, London, UK; 5 School of Biomedical Engineering and Imaging Sciences, King’s College London, London, UK; 6 Department of Radiation Safety and Medical Physics, Medical School Hannover, Hannover, Germany; 7 Institute for Medical Science and Technology IMSaT University Dundee, Dundee, UK

**Keywords:** PET-MRI, Motion correction, Phantom, MCFLIRT, BrainCompass, SIRF, SPM, NiftyReg

## Abstract

**Background:**

Due to comparatively long measurement times in simultaneous positron emission tomography and magnetic resonance (PET/MR) imaging, patient movement during the measurement can be challenging. This leads to artifacts which have a negative impact on the visual assessment and quantitative validity of the image data and, in the worst case, can lead to misinterpretations. Simultaneous PET/MR systems allow the MR-based registration of movements and enable correction of the PET data. To assess the effectiveness of motion correction methods, it is necessary to carry out measurements on phantoms that are moved in a reproducible way. This study explores the possibility of using such a phantom-based setup to evaluate motion correction strategies in PET/MR of the human head.

**Method:**

An MR-compatible robotic system was used to generate rigid movements of a head-like phantom. Different tools, either from the manufacturer or open-source software, were used to estimate and correct for motion based on the PET data itself (SIRF with SPM and NiftyReg) and MR data acquired simultaneously (e.g. MCLFIRT, BrainCompass). Different motion estimates were compared using data acquired during robot-induced motion. The effectiveness of motion correction of PET data was evaluated by determining the segmented volume of an activity-filled flask inside the phantom. In addition, the segmented volume was used to determine the centre-of-mass and the change in maximum activity concentration.

**Results:**

The results showed a volume increase between 2.7 and 36.3% could be induced by the experimental setup depending on the motion pattern. Both, BrainCompass and MCFLIRT, produced corrected PET images, by reducing the volume increase to 0.7–4.7% (BrainCompass) and to -2.8–0.4% (MCFLIRT). The same was observed for example for the centre-of-mass, where the results show that MCFLIRT (0.2–0.6 mm after motion correction) had a smaller deviation from the reference position than BrainCompass (0.5–1.8 mm) for all displacements.

**Conclusions:**

The experimental setup is suitable for the reproducible generation of movement patterns. Using open-source software for motion correction is a viable alternative to the vendor-provided motion-correction software.

**Supplementary Information:**

The online version contains supplementary material available at 10.1186/s40658-022-00442-6.

## Background

Simultaneous positron emission tomography and magnetic resonance (PET/MR) systems [1, 2] allow the registration of movements due to the high spatial and temporal resolution and the good contrast of the MR images [3, 4]. This makes it possible to correct the PET data, which is recorded simultaneously, e.g. regarding attenuation correction and patient motion [5–7]. There are several approaches regarding the correction of head movements during PET acquisition [8–18].

Due to the comparatively long measuring time in PET, patient movements are inevitable [19] and lead to artifacts, so-called blurring, which influence the imaging process. Movements, which can be divided into two types, further worsen the already comparatively low resolution of PET. In addition to strong, rapid movements of the head, there are also slow drift movements, which result, for example, from the relaxation of the patient’s muscles in the device [20]. Changes in the position of organs are also accompanied by movements of the lesions. This leads to misinterpretations of the tracer uptake, lesion size, decreased PET image quality and quantitative accuracy [21, 22]. Hence, applying some form of motion correction (MoCo) is recommended in PET studies [23]. The mentioned blurring effect leads to increase in the volume in lesions or in the case of this work of a flask in a phantom and serves as a measure of the quality of the MoCo.

Different methods for MoCo are possible, such as vendor-provided proprietary algorithms and custom offline solutions based on open-source software. The methods presented here use the echo-planar imaging (EPI) MR sequence for MoCo [21], which offers the possibility of registering movements with a high temporal resolution based on a rigid registration [24]. Alternatively, PET-only methods use non-attenuation corrected (NAC) or attenuation corrected (AC) images to estimate the motion [25–27]. Current methods for correcting movements in PET/MR show an improvement in PET image quality [4]. However, an evaluation of these procedures is difficult in a clinical setting using patient data. To evaluate such procedures, the registered movement in the PET/MR system must be known a priori and reproducible. This condition is not feasible for patient movement.

This study describes a phantom-based experimental setup for evaluation of novel procedures for MoCo. We have divided the paper into two parts, firstly the evaluation of two MoCo methods and a specially created motion-adapted algorithm for framing of the data, and secondly several algorithms were compared with regard to their tracking properties of the phantom movement.

## Methods

### Experimental setup

**Fig. 1:**
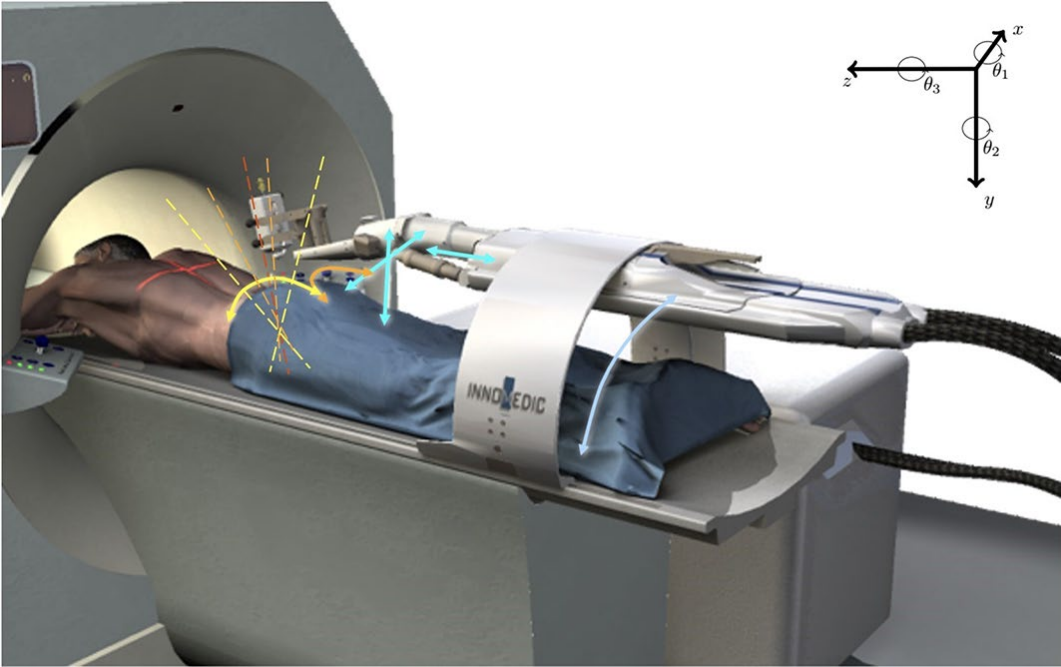
Schematic representation of the InnoMotion robotic system. The possible directions of movement are indicated by the coloured arrows. Translational movements were performed along the z-axis and rotations around the x-axis ($\theta _1$). Innomedic GmbH [28]

An MR-compatible robotic system (INNOMOTION, Innomedic GmbH, Herxheim, Germany, Fig. 1), which was initially developed to guide interventions, was used to generate rigid motion of a head phantom. The robot arm is pneumatically driven in 5 DOF (Degrees Of Freedom) [28]. Attached to a 180$^\circ$ ring, which can be fixed to the patient table, the arm can be manually prepositioned into the orbit region, at fixed angles [29]. Before taking a series of measurements, software-controlled initialisation and referencing was always carried out. The robotic arm has a maximum range of 150 mm in axial direction and a transverse range of 300 mm at holder (restricted by the magnet bore) and, according to manual, an accuracy of ± 1 mm and ± 1$^\circ$ when positioning.

**Fig. 2:**
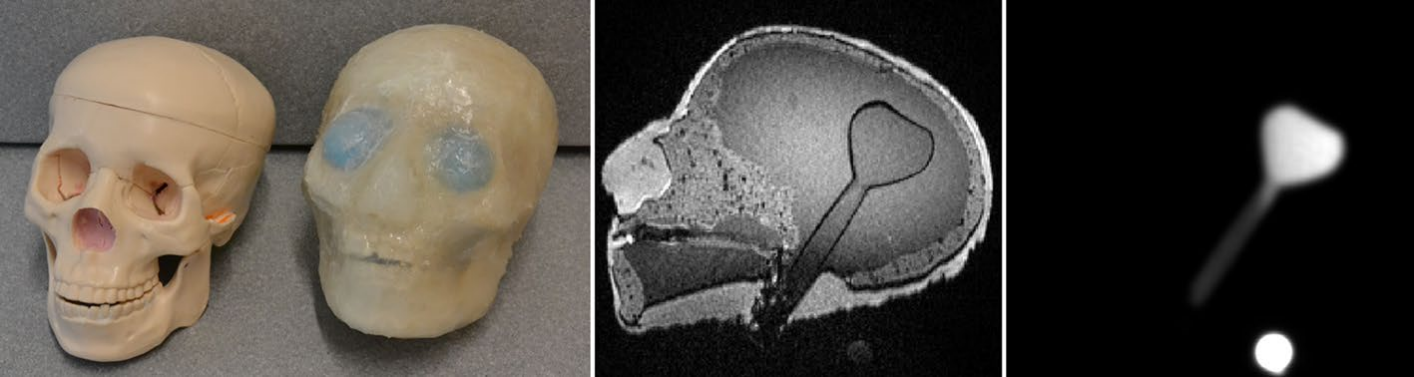
Commercial skull model and head phantom (left, [30]), corresponding $T_1$ MPRAGE of the head phantom (middle, for the sequence parameters see Table 1) and PET image of the filled flask within the phantom (right). The PET image (right) shows one of the three markers

**Fig. 3:**
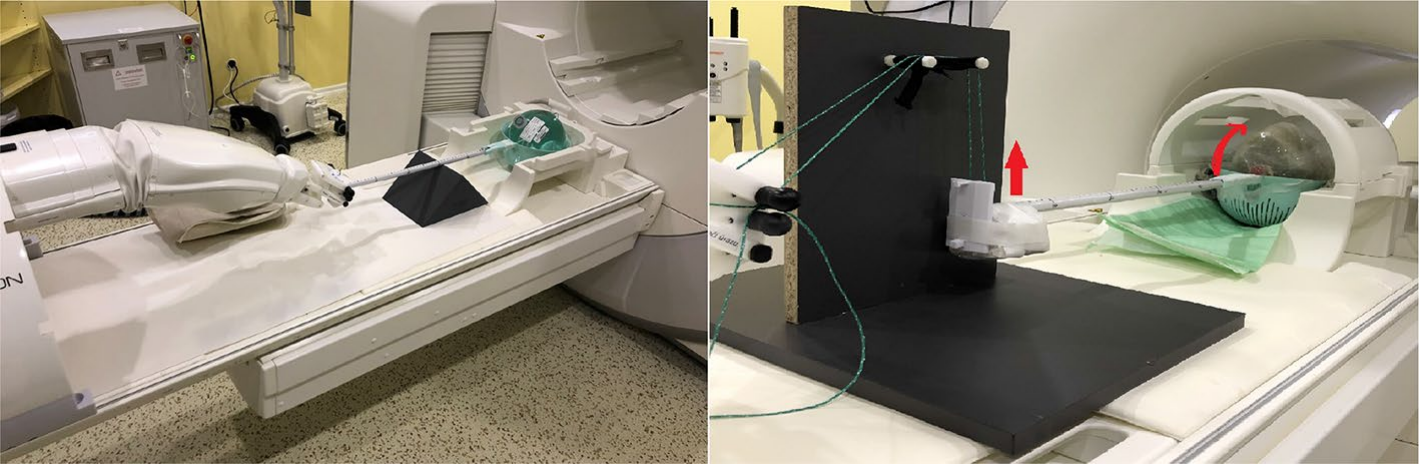
InnoMotion robot system and experimental setup for generating a translation motion (left). Extension of the setup to create rotations around the transverse axis (right). The pulling motion of the robot arm can be converted into a rotational motion (see red arrows)

A realistic head phantom [30] with an embedded lesion (fillable flask, 50 ml) was used (Fig. 2). This phantom was modeled to have a structure corresponding to the anatomy of the human skull, as well as attenuation and MR properties similar to human tissue. The phantom is equipped with a flask with a volume of 50 ± 0.12 ml at 20 $^\circ$C (VITLAB ©), which can be closed and is accessible from the outside. The flask is located in the brain region. For more information on this phantom (materials, construction and experimental evaluation), please refer to the relevant publication [30]. The flask was used as the basis for evaluating the quality of the MoCo processes. Since lesion movements result, among other consequences, in an increase in the resulting lesion volume, this was also used as an evaluation standard. The movement of the robotic arm could be transferred to the head phantom by a custom-made device consisting of plastic elements (Fig. 3).

It was possible to move the robot arm in z-direction (longitudinal axis) via the vendor-provided software (MotionCheck, V. 1.2.1.0). By extending the setup (Fig. 3), an additional rotational movement around the x-axis (transversal axis, head tilts towards or away from the chest; $\theta _1$ in Fig. 1) could be generated.

The movement was tracked using three markers (1.5 ml volume) mounted to the rigid experimental setup. The markers could be filled with a radioactive liquid for subsequent verification of the motion amplitude. By attaching them around the phantom, the same displacement affected the markers as the head phantom and they were visible in the 3D PET volume at the same time.

### Acquisition

Before starting a series of measurements, the flask within the phantom as well as the markers were filled with aqueous $^{18}$F-FDG (2-Fluoro-2-Deoxy-D-Glucose) solution. The activity was between 30 and 40 MBq at the time of the start of a measurement series. A measurement series (same motion amplitude) consisted of one static image without motion (reference measurement, reference volume), followed by four images taken under the same motion amplitude. This allowed the averaging over these four to estimate the stochastic error.

All measurements were performed using a Siemens Biograph mMR PET/MR (Siemens Healthineers, Erlangen, Germany). For the acquisition of the necessary data, an established clinical protocol was used (Table 1).

**Table 1:** Listing of the most important sequence parameters

Sequence (clinical)	Parameters	Values
MRAC Brain HiRes	Sequence TR TE Voxel size Flip-Angle Segmentation References	Dixon 4.14 ms 1.28/2.51 ms 2.1 $\times$ 2.1 $\times$ 2.0 mm$^3$ 10$^{\circ }$ fat, water, bone (built-in) [41]
EPI for MoCo	Sequence TR TE Voxel size Acquisitionmatrix Flip-Angle	EPI bold 2000 ms 30 ms 3.0 $\times$ 3.0 $\times$ 3.5 mm$^3$ 64 $\times$ 64 90$^{\circ }$
$T_1$ MPRAGE	Sequence TR TE Voxel size Acquisitionmatrix Flip-Angle	MPRAGE ($T_1$ weighted) 2400 ms 2.26 ms 1.0 $\times$ 1.0 $\times$ 1.0 mm$^3$ 256 $\times$ 256 8$^{\circ }$

The PET data were aquired in listmode (LM) format. The PET acquisition and EPI sequence ran simultaneously for 600 s each for all data sets. The following settings were used for the PET reconstruction: OSEM (8 iterations, 21 subsets), the Brain HiRes as $\mu$-Map, a relative scattering correction, a 256  $\times$  256 image matrix, 2.8 zoom and a Gauss-Filter with 3 mm FWHM. This resulted in a voxel size of 1.0 $\times$ 1.0 $\times$ 2.03 mm$^3$ after reconstruction.

### Data-processing methods

**Fig. 4:**
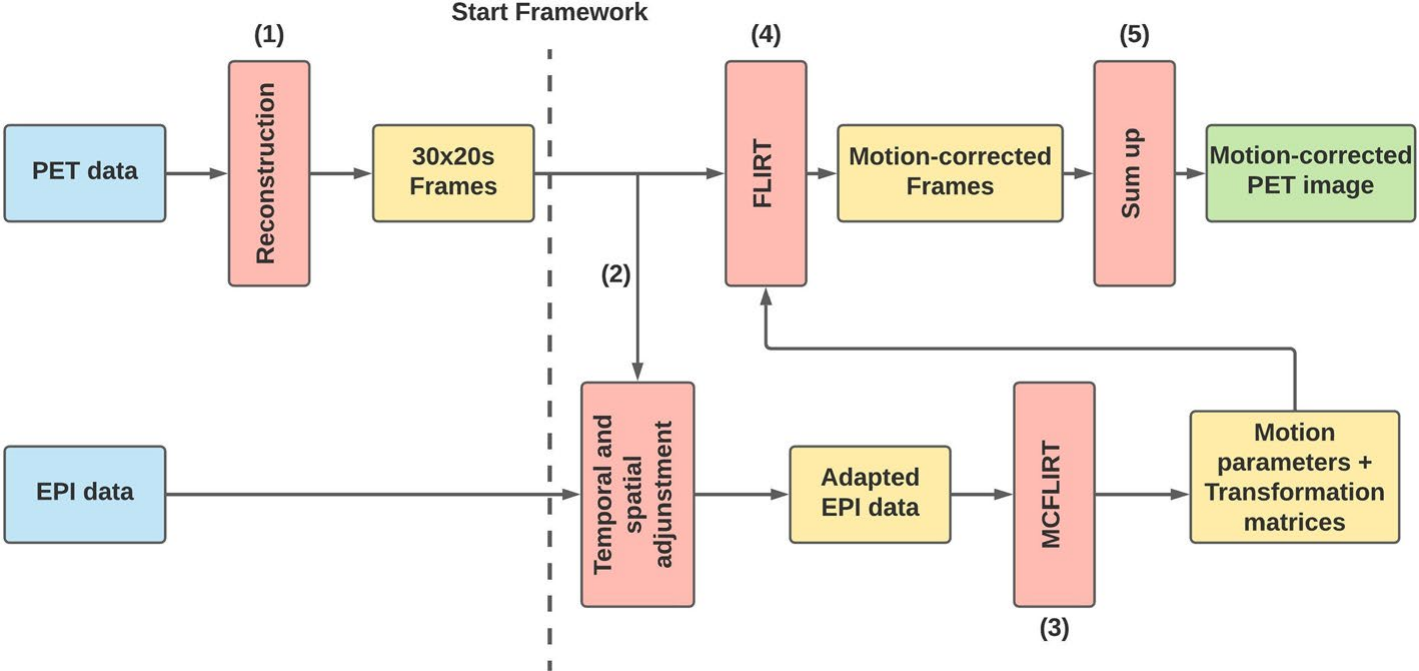
Framework for reconstruction of equidistant frames using MCFLIRT. (1) The PET data was split into frames and reconstructed by the manufacturer software (Siemens). (2) The MR data (EPI) were spatially and temporally adapted to the PET data. EPI data were interpolated by miconv (ODIN [35]) which uses an Akima spline [40] for this purpose. (3) MCFLIRT was executed based on the adapted EPI data. (4) The resulting transformation matrices are then applied to the PET frames. (5) Finally, all motion-corrected frames are summed

The following MoCo algorithms were evaluated: BrainCompass, which is part of the Biograph mMR, and MCFLIRT (FMRIB Software Library [31–33]). Datasets were reconstructed on the console into multiple frames without MoCo and into a single frame using BrainCompass. MCFLIRT was incorporated into the clinical routine via a framework (Fig. 4) to estimate the motion from the EPI images.

**Fig. 5:**
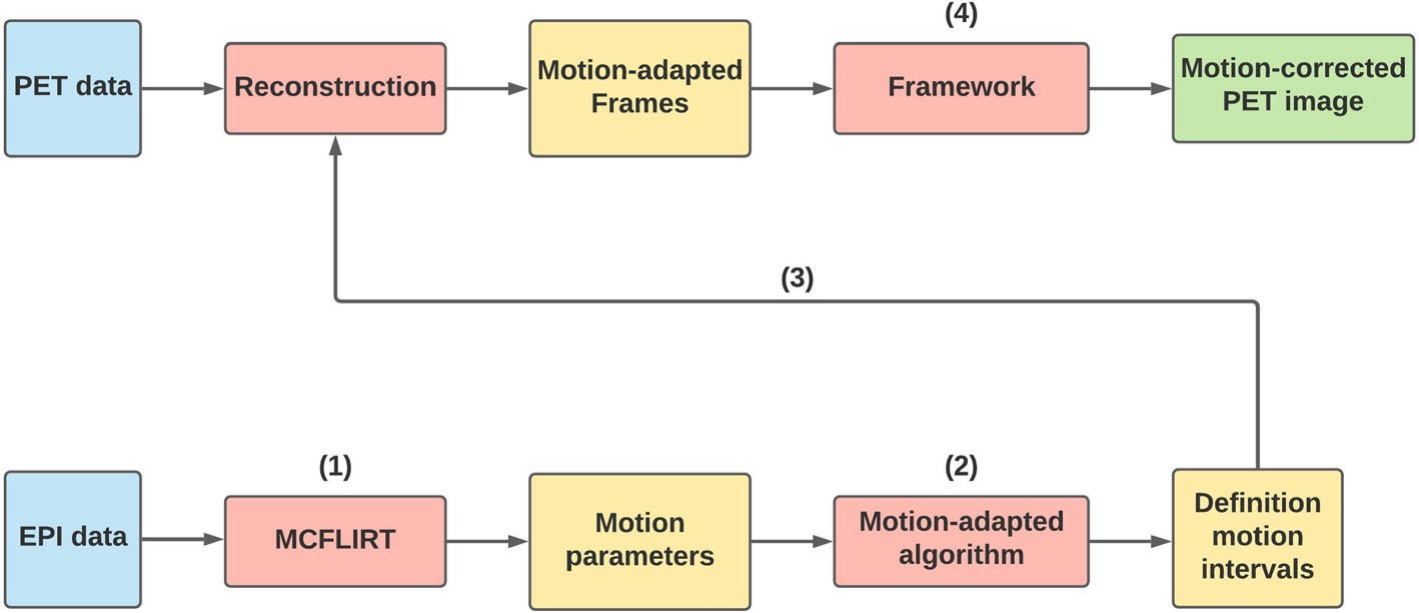
Framework for motion-adapted reconstruction using MCFLIRT. (1) MCFLIRT was applied to the EPI data. (2) An algorithm then determined the frames to be reconstructed. (3) Based on the defined movement intervals, the PET data were reconstructed on the Siemens console. (4) The framework from Fig. 4 was used to obtain a motion-corrected image

For MCFLIRT, different schemes for dividing the LM data into time frames were investigated. As a simple strategy, the PET data were divided into equidistant frames (30 $\times$ 20s frames). Alternatively, it is also possible to divide the PET measurement data into frames based on the motion that occurred [20]. The framework shown in Fig. 4 has been extended for this purpose (Fig. 5). The motion-adapted frame-splitting method detects sudden movements with an amplitude of more than 0.5 mm and uses the registered motion jumps as frame boundaries. The comparison is made using successive averaged translation values:

$$\begin{aligned} |d_{n}-d_{n-1}| \ge 0.5\, \text {mm} \end{aligned}$$

with $d_{n}$ is the absolute translation displacement calculated by MCFLIRT for the *n*-th EPI frame (2 s sampling). Frames of less than 20 s were appended to the previous frame (1$^{st}$ frame excluded) to avoid frames which are too short. To correct slow drift movements, a frame is prevented from lasting longer than 2 min by splitting the interval in the middle.

The default settings for all MCFLIRT-based results were trilinear interpolation and normalized correlation as cost function. These settings have not been changed for MoCo since they were found to provide best results in preliminary tests. To avoid any interference due to differences in PET reconstruction, the static volume (1st image of a measurement series) served as a reference for the volumes determined by MCFLIRT (motion-adapted frames and 30  $\times$  20s frames).

For the comparison of the registered motion, the motion parameters had to be extracted. MCFLIRT presents the motion parameters both in a (4 $\times$ 4)-matrix (.mat file) and in a list format (.par file) of the form rot$_{\alpha }$, rot$_{\beta }$, rot$_{\gamma }$, trans$_x$, trans$_y$, trans$_z$ per frame. The .par file was used for this work.

The BrainCompass, which uses the EPI sequence for motion correction, stores the motion parameters in the DICOM header in a separately created folder (MoCoSeries) for each dicom file ((0020,4000) LT [Motion: trans$_x$, trans$_y$, trans$_z$, rot$_{\alpha }$, rot$_{\beta }$, rot$_{\gamma }$]). The exact registration algorithm is not published in detail, but some information can be found in [34]. According to this, PET data is divided into individual motion states corresponding to the patient’s movement. The $\mu$-Map is adapted to the motion frames and after the reconstruction of all frames, a transformation to a reference PET frame takes place with subsequent summation.

### Movement patterns

Three types of motion patterns were generated for the evaluation of the different algorithms. Firstly, simple translational motions were generated along the z-axis (see Fig. 1) with different maximum motion amplitudes ranging from 0 to 20.24 mm. The single motion was performed 300 s after the start of acquisition, i.e., in the middle of the PET acquisition. Secondly, rotational movements around the x-axis ($\theta _1$ in Fig. 1), with rotation angles between 0$^\circ$ and 3.49$^\circ$, were generated. Again, the motion was initiated at 300 s. Finally, a more complex motion pattern compared to a simple translational motion was generated to verify motion-adapted frame-splitting (for illustration see Fig. 10) prior to MCFLIRT. The goal was to reach the final position by stepwise translational displacement along the z-axis. Eight steps of 0.5 mm translational displacement were performed consecutively, starting 180 s after the beginning of the PET acquisition. There were 20 s between each of the individual displacements. *Alternative 1* and *2* were generated as a result of the described method. In *Alternative 1*, the frame boundaries were set after each detected motion and the areas before and after the gradual movement (motion pattern) remained without further subdivision. *Alternative 2* allows a finer subdivision of the reconstructed frames even when no motion was detected. These different subdivisions were achieved by adjusting the motion-adapted algorithm by changing or removing the maximum time span of 2 min. A slow drift movement was not to be expected with the described phantom setup.

### Evaluation method

**Fig. 6:**
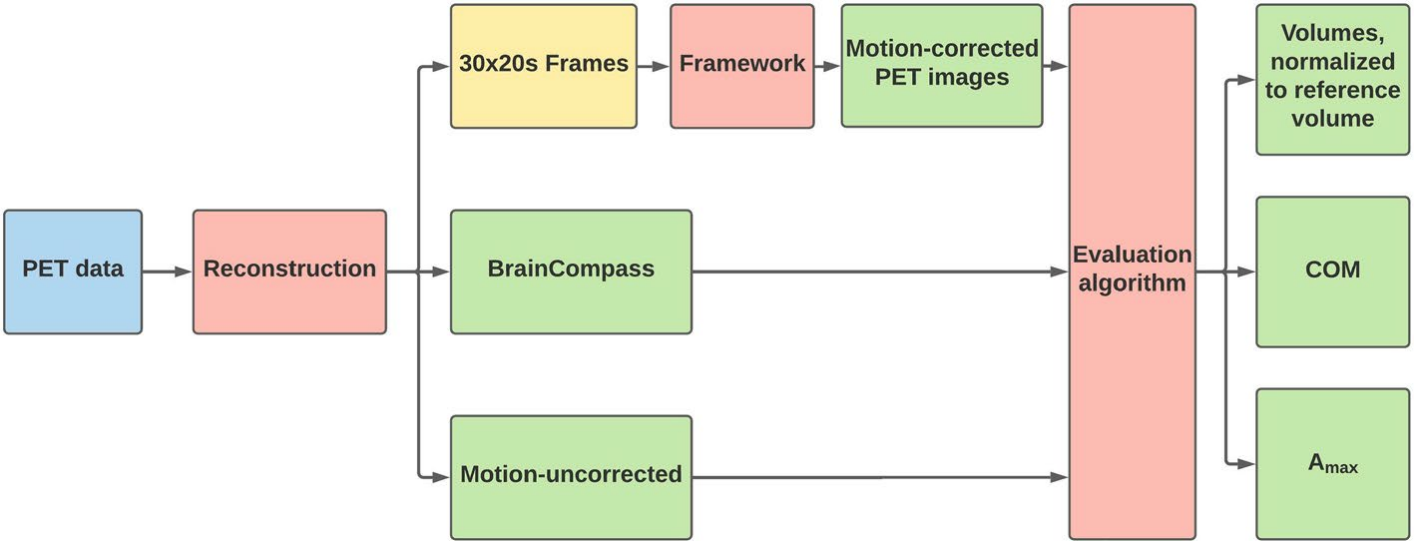
Schematic representation of the evaluation method. After reconstruction, three or four datasets were available: motion-uncorrected, BrainCompass and MCFLIRT (30 $\times$ 20s and if relevant motion-adapted). To obtain MCFLIRT corrected images, the frameworks of Figs. 4 and 5 had to be applied

For the evaluation of the datasets and the determination of the motion-corrupted and corrected volumes (comparative value), evaluation programs were written in Python and UNIX shell scripts. Furthermore, the Object-oriented Development Interface for NMR (ODIN version 2.0.4, [35]) was used to transform image data and perform arithmetic calculations with whole datasets. Four datasets were available for evaluation: the motion-uncorrected PET image, the motion-corrected PET images from BrainCompass and MCFLIRT with equidistant frames and, if relevant, motion-adapted frame splitting (Fig. 6). The following is a brief description of the procedure of the program. First, the markers had to be removed from the PET image using spherical masks. The maximum activity concentration $A_\text {max}$ and a lower threshold $0.1 \cdot A_\text {max}$ was then determined. Using this threshold, all voxels in the interval $[0.1 \cdot A_\text {max}, A_\text {max}]$ were summed up and the result characterized the volume. The lower threshold was selected based on preparatory evaluations which showed that the calculated volume was relatively insensitive to changes in the specific value of the threshold below 0.17. In a final step, all volumes were normalized to the reference volume (first measured value of a measurement series, not corrupted by movement). To check the plausibility of the movement generated by the robotic arm, it was possible to check the positions of the individual markers by a COM algorithm before and after the motion.

In the case of the motion-adapted reconstruction, the calculation time and memory requirements were compared with the MCFLIRT standard method (30 $\times$ 20s frames). To determine the calculation time, time stamps were incorporated into the framework and finally a difference was formed. The calculation time refers only to the registration and does not consider the reconstruction of the individual frames.

In addition to looking at volumes, the maximum activity concentration ($A_{max}$) and center-of-mass (COM) were used to evaluate the MoCo processes. The COM calculated on the basis of the PET image was determined before and after the motion, as well as after the application of BrainCompass and MCFLIRT. Similarly, $A_{max}$ was determined. ODIN was used to determine the COM and $A_{max}$ in each case. For the observation of $A_{max}$ it was necessary to perform a decay correction which was realized by a shell script using ODIN for each image.

### Comparison of different registration methods

Due to the well-known phantom motion, other registration methods could be considered in a comparison regarding motion tracking (see Figs. 11 and 12). Different motion estimates were compared with the robot-induced motion (ground truth). The open-source Synergistic Image Reconstruction Framework (SIRF) [36] was used to reconstruct PET images without attenuation correction (NAC) and estimate motion using SPM12 [37] and NiftyReg [38, 39]. Compared to MCFLIRT, the PET Raw Data in LM format were used here, so the workflow is independent of the Siemens software. During the reconstruction of the NAC images, the data were divided into the corresponding frames (30 $\times$ 20s or motion-adapted). The OSEM algorithm with 8 iterations and 21 subsets was used, taking into account randoms, detector sensitivity and scattering. This resulted in a voxel resolution of 2.32 $\times$ 2.32 $\times$ 2.03 mm$^3$ after reconstruction.

Furthermore, all algorithms (MCFLIRT, BrainCompass, SPM12 and NiftyReg) were compared with respect to registration based on EPI. The registrations based on EPI data have a temporal resolution of 2 s, while NAC-based registrations has used 30 $\times$ 20s frames. For comparability, the 1st frame was also defined as the reference frame here.

For comparison with MCFLIRT and BrainCompass, the motion parameters were saved in separate files. SIRF offers appropriate methods for saving the parameters in a (4 $\times$ 4)-matrix. From this, the rotation angles were calculated and the translation values extracted.

## Results

### Translation motion

**Fig. 7:**
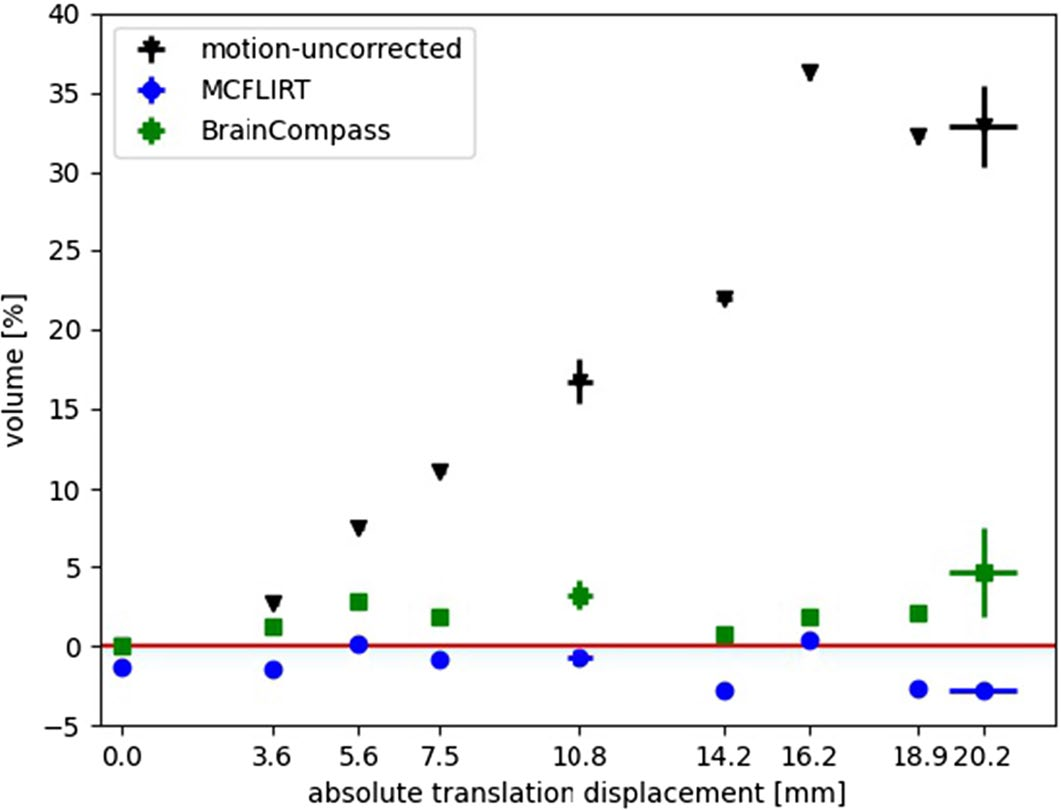
Results of MoCo procedures for different translation amplitudes by pulling the head phantom. Shown are the motion-uncorrected volume (black), BrainCompass (green) and MCFLIRT with equidistant frames (blue). The values were averaged over four identical motion amplitudes with the error bar in y-direction as the SD. The error bar in x-direction shows the fluctuations of the measured amplitude (SD)

Figure 7 shows results for an acquisition with several translations of different amplitudes in the longitudinal direction. The specified movement parameters all originate from MCFLIRT and have therefore been detected in the same coordinate system. Each manufacturer, each system and the various algorithms usually use different coordinate system origins (for PET and MR). This does not affect the absolute translation vector, but it does affect the registered rotation angles. To avoid errors here, the translation shift determined by MCFLIRT was always given as a “standard” measure. Thus, the results are comparable. The error with respect to the x-axis resulted from the standard deviation (SD) which was calculated by averaging over the absolute translation displacement of the measurement series. This can be caused by deviations in the execution of movements by the robotic arm or by deviations in the motion registration. The error bars that corresponds to the volumes are the SD with regard to the volume determination results.

For the motion-uncorrected case, the estimated volume of the reconstructed lesion (flask) increased as the motion amplitude increased. In contrast, BrainCompass and MCFLIRT corrected the movement. While BrainCompass volumes deviate from the reference volume by approximately 0.7–4.7%, the results of MCFLIRT are between  -2.8–0.4%. The horizontal red line by 100% in Fig. 7 corresponds to an ideal correction and that means a correction in which the reference volume would be reached.

**Fig. 8:**
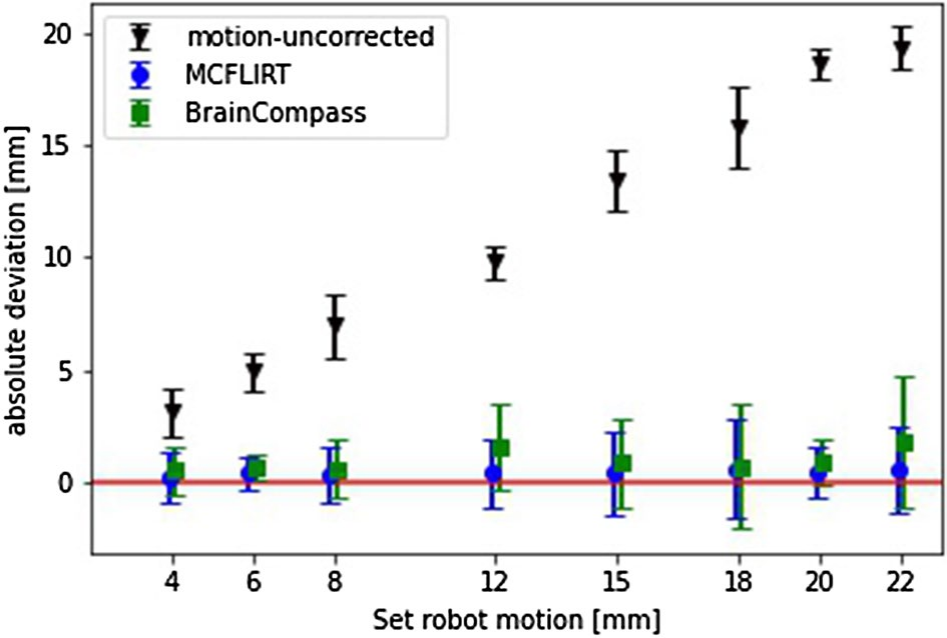
Representation of the absolute deviation of the COM without any MoCo and after MoCo using BrainCompass and MCFLIRT. The red line symbolizes 0 mm distance to the COM before the motion (reference). The values represent the Euclidean distance between two points in space

**Fig. 9:**
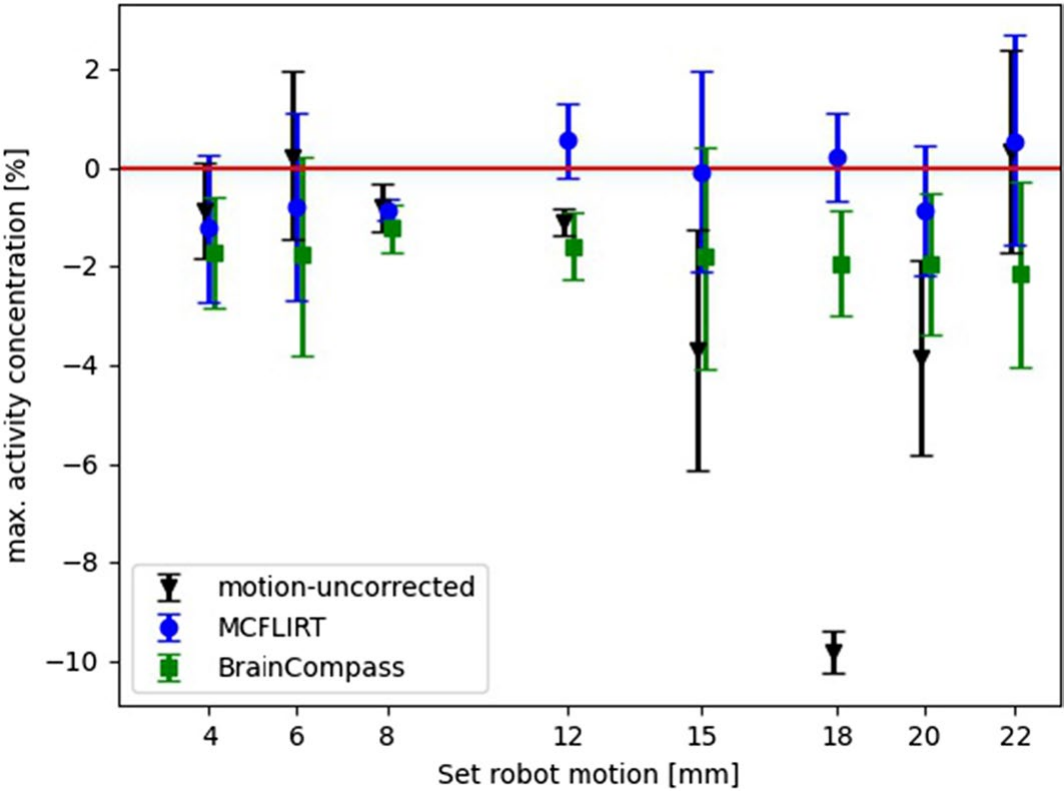
Representation of the relative deviation of $A_{max}$ without any MoCo and after application of BrainCompass and MCFLIRT. The red line symbolizes $A_{max}$ for the reference (before motion)

In order to evaluate the results using further assessment metrics, COM (Fig. 8 and the Additional file  1; Supplementary Table 1, 2) and $A_{max}$ (Fig. 9 and the Additional file  1; Supplementary Table 3) were also considered. Looking at the results for COM (Fig. 8), the distance (euclidean norm) from COM after applying BrainCompass and MCFLIRT to COM before the motion (reference position) is shown for the different set translation amplitudes. The results show that MCFLIRT (ranging from 0.2 to 0.6 mm after MoCo) had a smaller deviation from the reference position than BrainCompass (between 0.5 to 1.8 mm) for all examined displacements. Looking at the results for $A_{max}$ in Fig. 9 shows the relative deviation from the value before motion for motion-uncorrected, BrainCompass and MCFLIRT. The deviation of MCFLIRT from the reference value ($A_{max}$ before motion) is less than 1.2% for all translation amplitudes while the BrainCompass provides deviations between 1.3% and 2.2%.

**Fig. 10:**
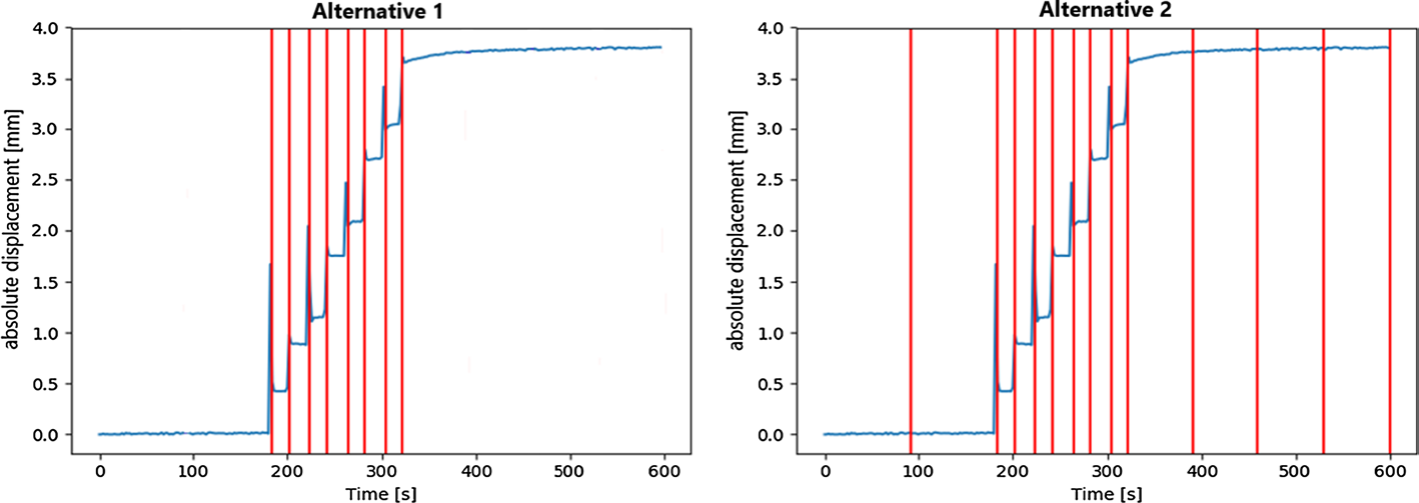
Schematic representation of the motion-adapted frames for *Alternative 1* (left) and for *Alternative 2* (right). The blue line represents the absolute translation amplitude and the red lines symbolize the frame boundaries

### Rotational motion

Results of BrainCompass and MCFLIRT for rotational movements are listed in Table 2. As expected, the motion-uncorrected volume increased with larger rotation angles, by 1.2–8.9%. However, while the corrected volume after BrainCompass increases with increasing rotation amplitude and for the 1.2$^\circ$ rotation even exceeds the motion-uncorrected volume, the volumes after MCFLIRT are closer to the reference volume.

**Table 2:** Volume increase (including SD) of MoCo methods for an increasing angle of rotation compared to the motion-uncorrected volume

Translation (mm)	Rotation (deg)	Motion-uncorrected (%)	BrainCompass (%)	MCFLIRT (%)
2.2 ± 0.1	1.2 ± 0.1	1.2 ± 0.3	2.9 ± 0.2	-0.2 ± 0.2
4.8 ± 0.4	2.6 ± 0.2	5.9 ± 0.6	3.9 ± 0.5	1.1 ± 0.3
5.9 ± 0.1	3.5 ± 0.2	8.9 ± 1.0	6.0 ± 1.1	1.2 ± 0.9

### Motion-adapted framing

A more complex motion pattern was generated to verify and evaluate the motion-adapted framing using MCFLIRT. Two different alternatives (referred as Alternative 1 and 2, Fig. 10) were created. While the motion pattern led to a relatively high volume increase of about 3.8% (Table 3) in the motion-uncorrected data, BrainCompass delivered an even larger volume of 3.9%. MCFLIRT, with a deviation of 1.2%, was closer to the reference volume. The alternatives showed also a similar deviation of 1.1% (A1ternative 1) and 1.2% (Alternative 2).

**Table 3:** Volume increase relative to the reference volume (with SD) for the motion pattern in Fig. 10

	Volume (%)
motion-uncorrected	3.8 ± 0.6
BrainCompass	3.9 ± 0.6
Standard (30 $\times$ 20s)	1.2 ± 0.7
Alternative 1	1.1 ± 0.5
Alternative 2	1.2 ± 0.6

**Table 4:** Calculation times and memory requirements for the different alternatives in Fig. 10

	Standard (30 $\times$ 20s)	Alternative 1	Alternative 2
Calculation time (s)	1146.3 ± 322.1	756.2 ± 127.6	587.8 ± 232.1
Memory (MB)	2048.3	942.9	679.6

Table 4 lists the time and memory required to calculate the motion corrected images with the different subdivisions using MCFLIRT.

### Comparison of different registration methods

**Fig. 11:**
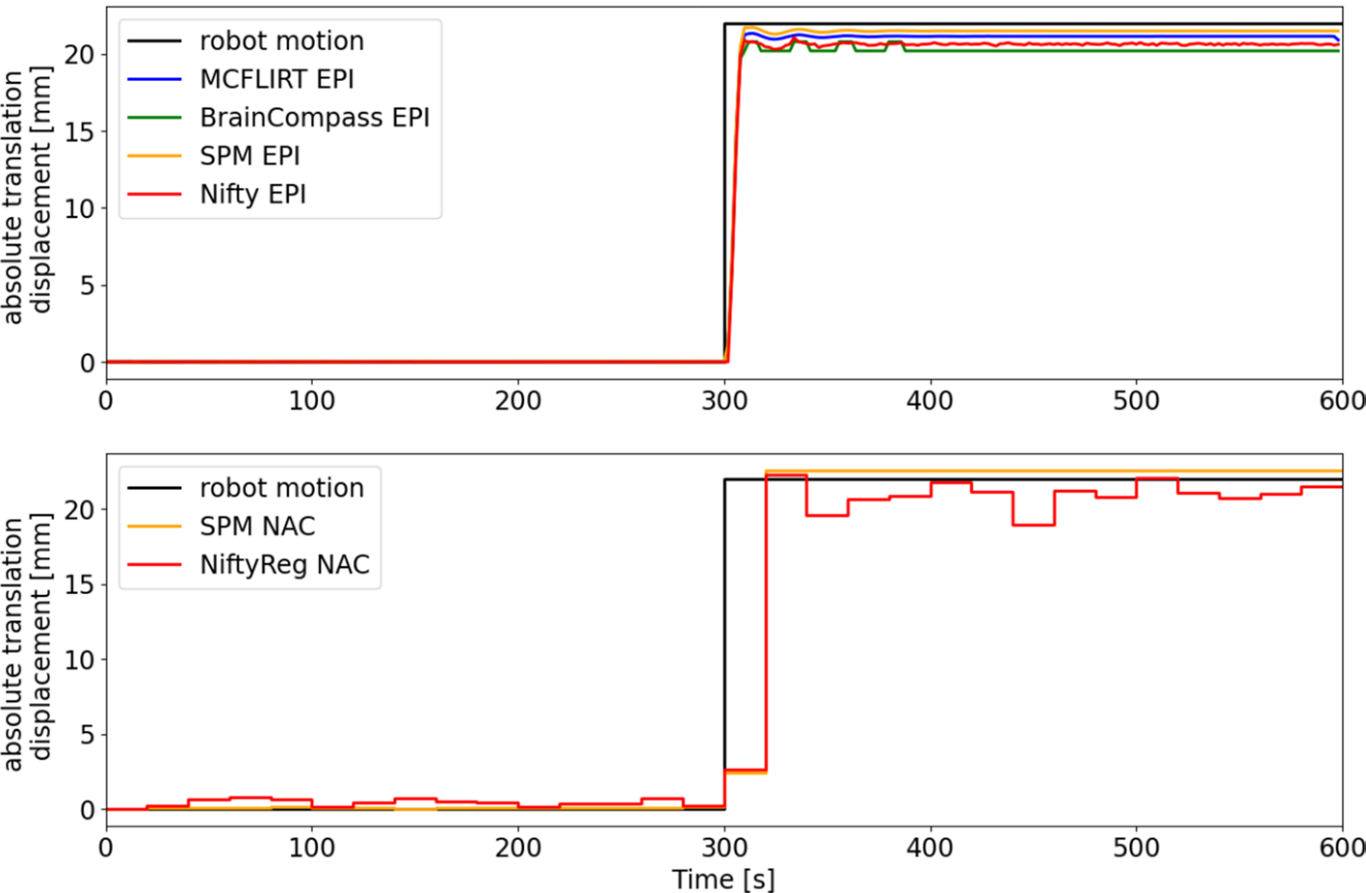
Comparison of different registration methods on a single translation movement. The robotic movement (black line) symbolizes the ground truth. Upper image: EPI-based; lower image: NAC-based. Shown is the absolute translation displacement depending on time

**Fig. 12:**
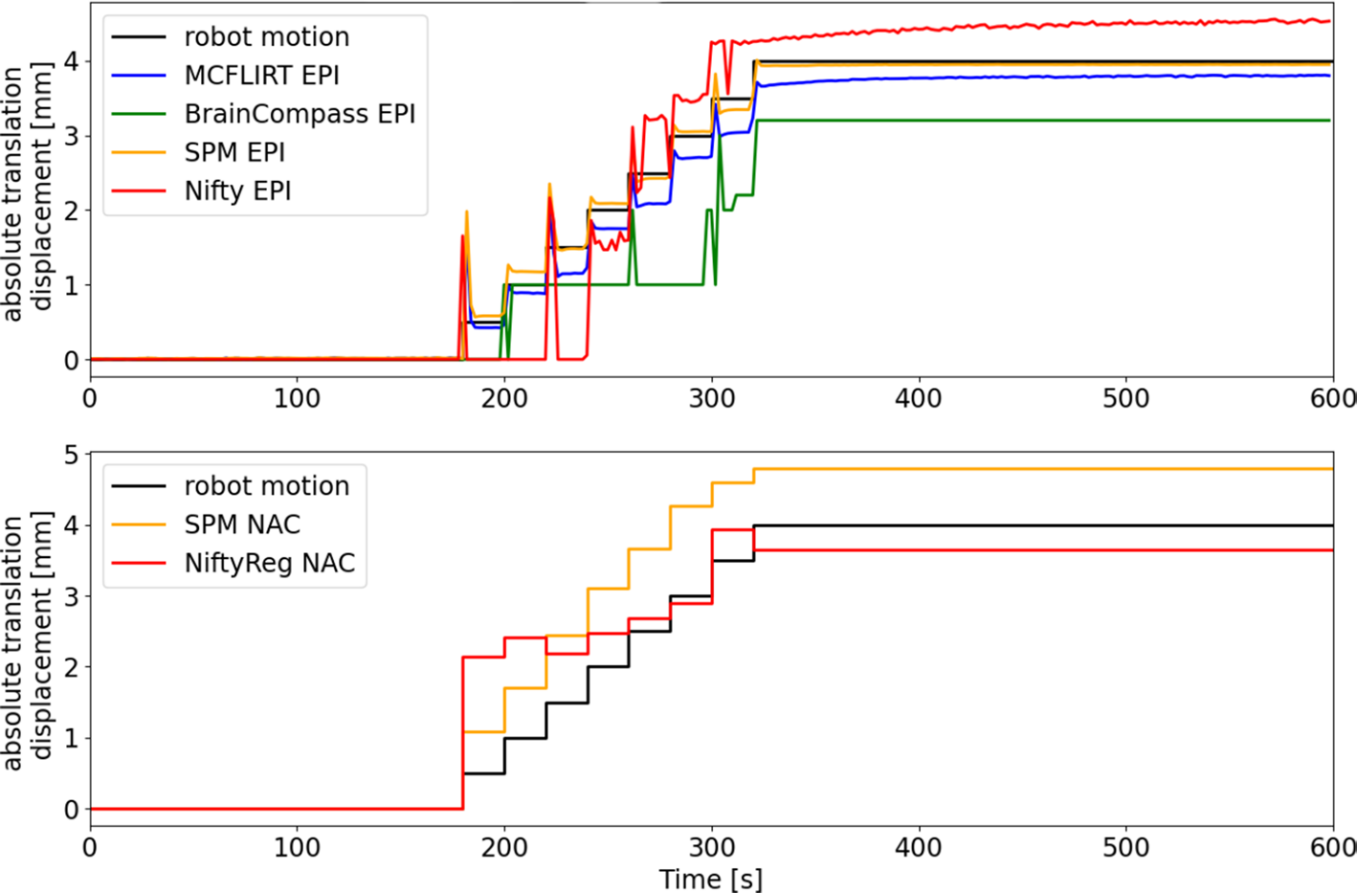
Comparison of different registration methods of multiple translation movements. The robotic movement (black line) symbolizes the ground truth. Upper image: EPI-based; lower image: NAC-based. Shown is the absolute translation displacement depending on time

Finally, various registration methods were tested based on a single large translation movement (Fig. 11) and on the motion pattern (Fig. 12). The given course of movement of the robotic system served as reference value (ground truth). In the upper plots the different registration methods are compared regarding EPI images, in the lower plots regarding NAC images.

In the upper plot of Fig. 11, all registration methods showed similar results. It shows very small discrepancies between MCFLIRT, BrainCompas, SPM and NiftyReg (all EPI-based). The deviation from the ground truth never exceeds 0.5 mm. In the lower image, the registrations based on the NAC images detected the maximum motion amplitude, but showed a slight fluctuation.

Figure 12 shows that the EPI-based results detected the individual movement steps. The deviations between the ground truth and MCFLIRT or SPM12 remained smaller than 0.5 mm. Only for the motion peaks the difference was larger than 0.5 mm. The BrainCompass could not detect all motion steps and underestimated the maximum displacement by about 1 mm. NiftyReg detected the movement, but overestimated the maximum translation displacement. In the NAC-based methods, it was found that SPM12 overestimated the maximum displacement, while NiftyReg detected a too low amplitude.

## Discussion

Compared to similar recent publications, e.g. [15, 17], a more realistic phantom (with bone analogues and anatomical structures) was used in this study so that motion-correction methods can be evaluated with reduced error in attenuation correction and/or without the need for synthetic $\mu$-Maps. Furthermore, several translational and rotational movements were recorded. The use of an MR-compatible robotic system, which was initially intended for interventions, made it possible to generate reproducible real-world (i.e. non-synthetic) movement sequences. The aim of our work was to evaluate different algorithms. For this purpose, the BrainCompass and a MCFLIRT framework, which were integrated into the clinical workflow, were compared. Using SIRF, it was subsequently possible to compare EPI- and NAC-based approaches using SPM12 and NiftyReg with regard to movement registration.

### Comparison of BrainCompass and MCFLIRT

When analyzing Fig. 7, Tables 2 and 3 , it becomes apparent that, in the motion-uncorrected case, the volume increases with increasing movement amplitude. This was expected because the activity concentration is smeared over a larger volume due to motion. Both BrainCompass and MCFLIRT corrected the volume, with MCFLIRT providing results closer to 100% of the reference volume. The corrected volumes differ between BrainCompass and MCFLIRT by approximately 1.3-7.4%. At this point, it should be noted that the exact algorithm of the BrainCompass image reconstruction is proprietary, and the cost function, optimization algorithm and interpolation that are employed are generally unknown to the end user.

The inspection of the COM (Fig. 8) shows that both BrainCompass and MCFLIRT corrected the flask to its original position (reference position before motion). While the results of the COM confirm the results from the volume analysis, the results regarding $A_{max}$ (Fig. 9) show only a small change in $A_{max}$ after motion. The reason for this was the overlap of the volume before and after the motion. The flask is about 5 cm in diameter at the thickest point, and the volumes overlap in the resulting 3D image when moving up to a maximum of 20 mm. Thus, a MoCo by BrainCompass or MCFLIRT did not provide any improvement with respect to $A_{max}$. It is striking to look at the results for the adjusted 18 mm displacement, this value is also visible in Fig. 7 (uncorrected). The reason was probably insufficient mixing of FDG and water prior to the measurement series at 18 mm. Thus, in the reconstructed image, there is an increased concentration of activity in the upper part, which leads to a bigger impact due the motion, as the translation amplitude was greater than the extent of activity accumulation. However, the MoCo methods also show an improvement here in the form of the restoration of $A_{max}$.

To check the motion-adapted frame-splitting algorithm (Fig. 5) for its suitability, a motion pattern (Fig. 10) was designed and applied. The algorithm detected the individual amplitude jumps (movements) and set them as frame boundaries. The volumes (Table 3) based on the motion-adapted subdivision were nearly identical to the standard subdivision (30 $\times$ 20s frames). Furthermore, the BrainCompass showed no improvement compared to the motion-uncorrected volume.

The duration of the calculations and the memory requirements were also compared (Table 4). As expected, with a lower number of frames, the calculation time as well as the storage space requirement decreases. This is due to the reduced number of required calculations (time saving) and fewer intermediate results (memory space saving). Furthermore, the occurrence of strong movements within a frame is avoided by motion-adapted frame splitting. This is supposed to prevent the blurring effect from occurring within a frame, which makes the calculation of motion parameters more accurate at least with regard to a NAC-based registration.

The direct comparison of both correction methods shows that both, BrainCompass and MCFLIRT, deliver motion-corrected images. One advantage of MCFLIRT is its open-source license. However, to obtain the results in the presented format, it was necessary to integrate the MCFLIRT into the clinical routine via a framework, in the form of a DICOM node. This is opposed by BrainCompass, an algorithm distributed by Siemens, which is available as part of the PET/MR system, i.e. it is easy to use and certified. The disadvantages are, on the one hand, the necessity of a proprietary license and, on the other hand, the missing information of the exact algorithm and the missing possibility of modifying settings like interpolation or cost function. Both algorithms functioned during all measurements without bugs, failures or similar.

### Comparison of different registration methods

In the Figs. 11 and 12 the motion registration hardly differs between MCFLIRT and SPM (EPI-based). Figure 11 shows that the registration using the NAC images (NiftyReg and SPM) is almost identical to the ground truth, with some fluctuations, likely due to the poor SNR on which the registration is based. It should be taken into account that a larger volume (brain image) was available for EPI-based registration, but PET-based could only use the flask and markers for registration.

Due to the better temporal resolution of the EPI data, peaks can be seen in the EPI motion tracking (Figs. 10 and 12), which were the result of movements when the phantom moved. The PET image or the individual frames weren’t affected by these spikes, which can also be seen in the course of the registrations (lower plots in Fig. 12) based on the NAC images. This is because the temporal resolution of the PET is not high enough to register this short movement.

### Limitations of the study

There are several limitations regarding this study: The whole setup (robotic system, phantom) represents a simplification of the actual real-world problem of motion correction of a brain measurement. In the context of this work, only simple motion patterns were used to evaluate complex algorithms. However, patient movement is usually more complex, a point that could not be fully reproduced here. For instance, a continuous slow spatial drift, which is often observed in patients, could not be created with the robotic system. The software of the robotic system (MotionCheck, V. 1.2.1.0) does not allow continuous motion. The anatomy in the phantom is also simple, the flask is well defined on the MR and the PET so there is no anatomic and/or physiological background unlike in a real patient. This limitation is expected to impact the accuracy of the NAC registrations, compared to patient data for radiotracers with distributed uptake in the brain.

## Conclusions

The experimental setup is suitable for the reproducible generation of movement patterns. This enabled an evaluation of rigid MoCo methods. In addition to the proprietary software BrainCompass, the open-source software MCFLIRT is a suitable alternative. The evaluation showed that MoCo methods lead to a minimization of volume increase through motion, which may result in a better localization of PET data. Furthermore, an algorithm for motion-adapted reconstruction was presented. The program is primarily used for MoCo that can be adapted to the patient’s movements and thus extends MoCo using MCFLIRT.

Registration quality has been demonstrated with both SPM and NiftyReg, as provided by SIRF. Future work could therefore include a comparison of motion-corrected PET reconstructions using SIRF.

## Supplementary Information

**Additional file 1. Supplementary Table 1.** The table shows the position (z,y,x) of the COM before and after the motion. The 1st column shows the originally set movement amplitude along the z-axis. **Supplementary Table 2.** The table shows the position (z,y,x) of the COM after application of BrainCompass and MCFLIRT. The 1st column shows the originally set movement amplitude along the z-axis. **Supplementary Table 3.** $A_{max}$ in [Bq/ml] reference (before motion), without any MoCo (motion-uncorrected) and after application of BrainCompass and MCFLIRT. The 1st column shows the originally set movement amplitude along the z-axis.

## Data Availability

The datasets generated during and/or analysed during the current study are available from the corresponding author on reasonable request.
