# Supplementary material for: Evaluating different methods of MR-based motion correction in simultaneous PET/MR using a head phantom moved by a robotic system
Source: EJNMMI Phys. 2022 Mar 3;9:15. doi: 10.1186/s40658-022-00442-6 (PMC8894542; doi:10.1186/s40658-022-00442-6)
Supplement: Supplementary file 1 — Additional file 1. Supplementary Table 1. The table shows the position (z,y,x) of the COM before and after the motion. The 1st column shows the originally set movement amplitude along the z-axis. Supplementary Table 2. The table shows the position (z,y,x) of the COM after application of BrainCompass and MCFLIRT. The 1st column shows the originally set movement amplitude along the z-axis. Supplementary Table 3. $A_{max}$Amax in [Bq/ml] reference (before motion), without any MoCo (motion-uncorrected) and after application of BrainCompass and MCFLIRT. The 1st column shows the originally set movement amplitude along the z-axis. [file 40658_2022_442_MOESM1_ESM.docx]

**Table 1**

The table shows the position (z,y,x) of the COM before and after the motion. The 1st column shows the originally set movement amplitude along the z-axis.

| Set robot motion (mm) | COM | | | | | |
| --- | --- | --- | --- | --- | --- | --- |
|  | Before motion | | | After motion | | |
|  | $z\pm SD$ | $y\pm SD$ | $x\pm SD$ | $z\pm SD$ | $y\pm SD$ | $x\pm SD$ |
| 4 | 50.4 $\pm$ 0.3 | 195.0 $\pm$ 0.1 | 131.7 $\pm$ 0.3 | 48.9 $\pm$ 0.3 | 195.0 $\pm$ 0.1 | 131.6 $\pm$ 0.3 |
| 6 | 51.8 $\pm$ 0.2 | 195.2 $\pm$ 0.2 | 130.4 $\pm$ 0.1 | 49.4 $\pm$ 0.2 | 195.3 $\pm$ 0.2 | 130.8 $\pm$ 0.1 |
| 8 | 52.5 $\pm$ 0.2 | 195.7 $\pm$ 0.1 | 135.0 $\pm$ 0.6 | 49.0 $\pm$ 0.3 | 195.9 $\pm$ 0.2 | 135.0 $\pm$ 0.7 |
| 12 | 53.8 $\pm$ 0.5 | 198.8 $\pm$ 0.2 | 134.4 $\pm$ 0.3 | 49.0 $\pm$ 0.2 | 198.7 $\pm$ 0.1 | 134.5 $\pm$ 0.2 |
| 15 | 55.6 $\pm$ 0.3 | 196.3 $\pm$ 0.1 | 136.5 $\pm$ 1.1 | 48.9 $\pm$ 0.3 | 196.7 $\pm$ 0.1 | 136.3 $\pm$ 0.7 |
| 18 | 57.0 $\pm$ 0.3 | 198.5 $\pm$ 0.3 | 132.5 $\pm$ 1.8 | 49.2 $\pm$ 0.3 | 198.7 $\pm$ 0.2 | 133.3 $\pm$ 1.0 |
| 20 | 54.4 $\pm$ 0.2 | 198.0 $\pm$ 0.1 | 135.7 $\pm$ 0.4 | 45.3 $\pm$ 0.2 | 198.6 $\pm$ 0.1 | 135.0 $\pm$ 0.2 |
| 22 | 55.4 $\pm$ 0.5 | 195.9 $\pm$ 0.1 | 132.8 $\pm$ 0.7 | 45.9 $\pm$ 0.2 | 196.4 $\pm$ 0.1 | 133.0 $\pm$ 0.4 |

**Table 2**

The table shows the position (z,y,x) of the COM after application of BrainCompass and MCFLIRT. The 1st column shows the originally set movement amplitude along the z-axis.

| Set robot motion (mm) | COM | | | | | |
| --- | --- | --- | --- | --- | --- | --- |
|  | Before motion | | | After motion | | |
|  | $z\pm SD$ | $y\pm SD$ | $x\pm SD$ | $z\pm SD$ | $y\pm SD$ | $x\pm SD$ |
| 4 | 50.2 $\pm$ 0.3 | 195.3 $\pm$ 0.1 | 131.7 $\pm$ 0.3 | 50.5 $\pm$ 0.3 | 195.2 $\pm$ 0.1 | 131.7 $\pm$ 0.3 |
| 6 | 51.6 $\pm$ 0.2 | 195.7 $\pm$ 0.2 | 130.5 $\pm$ 0.1 | 51.9 $\pm$ 0.2 | 195.4 $\pm$ 0.2 | 130.6 $\pm$ 0.1 |
| 8 | 52.3 $\pm$ 0.3 | 196.2 $\pm$ 0.2 | 135.0 $\pm$ 0.7 | 52.6 $\pm$ 0.3 | 196.0 $\pm$ 0.1 | 135.0 $\pm$ 0.6 |
| 12 | 53.3 $\pm$ 0.6 | 199.9 $\pm$ 0.4 | 134.3 $\pm$ 0.3 | 53.9 $\pm$ 0.5 | 199.0 $\pm$ 0.2 | 134.3 $\pm$ 0.3 |
| 15 | 55.3 $\pm$ 0.3 | 197.0 $\pm$ 0.2 | 136.6 $\pm$ 1.0 | 55.7 $\pm$ 0.4 | 196.6 $\pm$ 0.1 | 136.5 $\pm$ 1.0 |
| 18 | 56.7 $\pm$ 0.3 | 199.1 $\pm$ 0.4 | 132.6 $\pm$ 1.9 | 57.1 $\pm$ 0.2 | 198.8 $\pm$ 0.3 | 133.0 $\pm$ 1.5 |
| 20 | 54.1 $\pm$ 0.2 | 198.6 $\pm$ 0.1 | 135.8 $\pm$ 0.4 | 54.5 $\pm$ 0.2 | 198.3 $\pm$ 0.4 | 135.5 $\pm$ 0.4 |
| 22 | 54.8 $\pm$ 0.7 | 197.2 $\pm$ 0.9 | 132.6 $\pm$ 0.7 | 55.6 $\pm$ 0.5 | 196.3 $\pm$ 0.1 | 132.7 $\pm$ 0.7 |

**Table 3**

$A_{\max}$ in [Bq/ml] reference (before motion), without any MoCo (motion-uncorrected) and after application of BrainCompass and MCFLIRT. The 1st column shows the originally set movement amplitude along the z-axis.

| Set robot motion (mm) | $A_{max}$ (Bq/ml) | | | |
| --- | --- | --- | --- | --- |
|  | Reference | Motion-uncorrected | BrainCompass | MCFLIRT |
|  | $A_{max}\pm SD$ | $A_{max}\pm SD$ | $A_{max}\pm SD$ | $A_{max}\pm SD$ |
| 4 | 363408.8 $\pm$ 3484.2 | 360193.8 $\pm$ 3535.9 | 357040.0 $\pm$ 3974.7 | 358888.8 $\pm$ 5341.3 |
| 6 | 352971.8 $\pm$ 8111.4 | 353795.0 $\pm$ 6093.9 | 346626.0 $\pm$ 6989.7 | 350102.0 $\pm$ 6666.5 |
| 8 | 353639.3 $\pm$ 3909.7 | 350721.5 $\pm$ 1737.4 | 349214.3 $\pm$ 1693.3 | 350490.0 $\pm$ 740.5 |
| 12 | 397528.0 $\pm$ 3266.7 | 393027.8 $\pm$ 1117.7 | 391122.5 $\pm$ 2615.9 | 399724.8 $\pm$ 3009.6 |
| 15 | 434349.8 $\pm$ 9221.6 | 418280.8 $\pm$ 10135.2 | 426363.0 $\pm$ 9575.5 | 433983.9 $\pm$ 8822.9 |
| 18 | 524773.0 $\pm$ 5394.8 | 473230.5 $\pm$ 2029.9 | 514469.3 $\pm$ 5451.7 | 525853.8 $\pm$ 4747.9 |
| 20 | 312934.3 $\pm$ 5032.9 | 300898.0 $\pm$ 5899.8 | 306757.3 $\pm$ 4357.9 | 310166.8 $\pm$ 4153.5 |
| 22 | 336636.8 $\pm$ 5386.5 | 337700.8 $\pm$ 6919.4 | 329359.0 $\pm$ 6164.6 | 338451.0 $\pm$ 7173.2 |
